# Mitochondrial Dysfunction in Repeat Expansion Diseases

**DOI:** 10.3390/antiox12081593

**Published:** 2023-08-10

**Authors:** Alberto Giménez-Bejarano, Eva Alegre-Cortés, Sokhna M. S. Yakhine-Diop, Patricia Gómez-Suaga, José M. Fuentes

**Affiliations:** 1 Departamento de Bioquímica y Biología Molecular y Genética, Facultad de Enfermería y Terapia Ocupacional, Universidad de Extremadura, 10003 Cáceres, Spain; agimenezb@unex.es (A.G.-B.); evalegrec@unex.es (E.A.-C.); smsyakhinediop@unex.es (S.M.S.Y.-D.); pgomezsuaga@unex.es (P.G.-S.); 2 Centro de Investigación Biomédica en Red en Enfermedades Neurodegenerativa, Instituto de Salus Carlos III (CIBER-CIBERNED-ISCIII), 28029 Madrid, Spain; 3 Instituto de Investigación Biosanitaria de Extremadura (INUBE), 10003 Cáceres, Spain

**Keywords:** Huntington disease, *C9orf72*, myotonic dystrophy type 1, Ca^2+^, mitophagy, apoptosis, ROS

## Abstract

Repeat expansion diseases are a group of neuromuscular and neurodegenerative disorders characterized by expansions of several successive repeated DNA sequences. Currently, more than 50 repeat expansion diseases have been described. These disorders involve diverse pathogenic mechanisms, including loss-of-function mechanisms, toxicity associated with repeat RNA, or repeat-associated non-ATG (RAN) products, resulting in impairments of cellular processes and damaged organelles. Mitochondria, double membrane organelles, play a crucial role in cell energy production, metabolic processes, calcium regulation, redox balance, and apoptosis regulation. Its dysfunction has been implicated in the pathogenesis of repeat expansion diseases. In this review, we provide an overview of the signaling pathways or proteins involved in mitochondrial functioning described in these disorders. The focus of this review will be on the analysis of published data related to three representative repeat expansion diseases: Huntington’s disease, *C9orf72*-frontotemporal dementia/amyotrophic lateral sclerosis, and myotonic dystrophy type 1. We will discuss the common effects observed in all three repeat expansion disorders and their differences. Additionally, we will address the current gaps in knowledge and propose possible new lines of research. Importantly, this group of disorders exhibit alterations in mitochondrial dynamics and biogenesis, with specific proteins involved in these processes having been identified. Understanding the underlying mechanisms of mitochondrial alterations in these disorders can potentially lead to the development of neuroprotective strategies.

## 1. Introduction

### 1.1. Repeat Expansion Diseases

**Figure 1:**
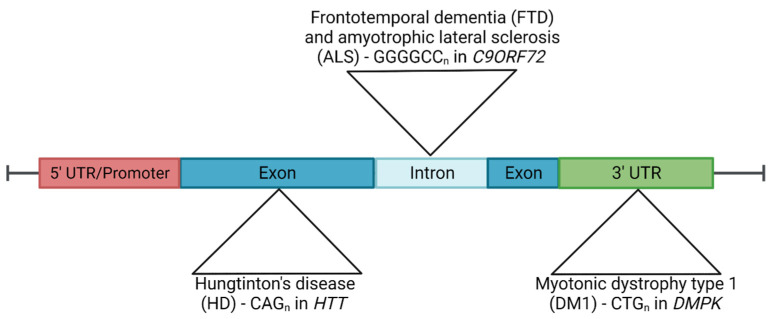
Diagram of the location of the expanded repeats in HD, *C9orf72* FTD/ALS and DM1. HD is caused by a CAG expansion in exon 1 of the HTT gene. The *C9orf72* mutation consists in a GGGGCC HRE in the first intron of the gene responsible for the most common cause of familiar forms of FTD and ALS. A CTG trinucleotide found in the 3’ untranslated region (UTR) of the DMPK gene results in the development of DM1.

**Figure 2:**
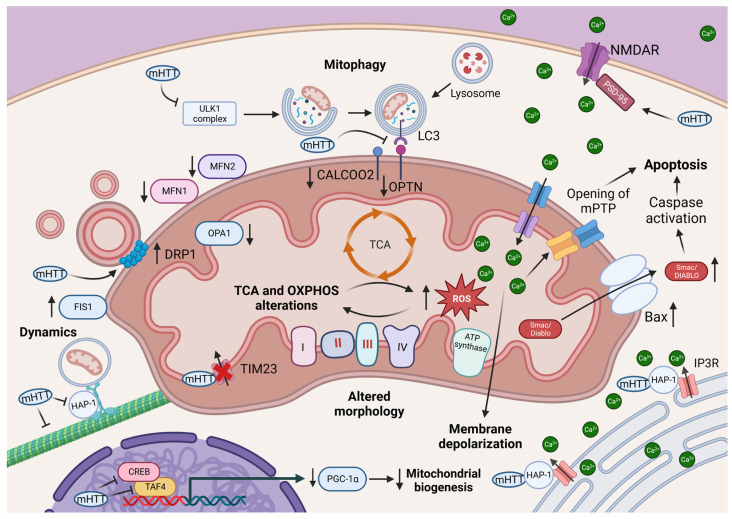
Mitochondrial alterations associated with HD. mHTT interaction with TIM23 hinders mitochondrial protein trafficking. A reduction in the complexes (I to IV) of the electron transport chain (ETC) such as complexes II and III activity has been reported in HD. TCA enzymes such as aconitase, α-KGDH and SDH activities are also altered in HD mice and human brain samples. These changes affect ROS generation, incrementing their levels. Mitochondrial fusion and fission processes are imbalanced, whit a rise in pro-fission proteins DRP1 and FIS1 and a decrease in pro-fusion proteins MFN1, MFN2 and OPA1. mHTT can interact with DRP1, resulting in fragmented mitochondria. mHTT may additionally interfere with CREB and TAF4 transcription factors, reducing PGC-1α levels and function. Moreover, vesicular transport is hindered by the interaction between mHTT, HAP-1 and microtubules. NMDAR are overactivated due to the lack of binding between mHTT and PSD-95. Moreover, mHTT interacts with IP3R in the ER. All this results in an increase in Ca^2+^ cytosolic concentration, which is buffered by mitochondrial Ca^2+^ uptake. The increase in Ca^2+^ can be responsible of the membrane depolarization observed in HD mitochondria. Mitophagy initiation complex formation and LC3-II binding to SQSTM1, CALCOCO2 and OPTN are disrupted by mHTT. In addition, there have been reports of increased Smac/DIABLO release from mitochondria, elevated levels of Bax and more caspase activity in HD, leading to a rise in apoptosis.

The discovery in 1991 of augmented versions of CGG repeats in the FMR1 gene and expanded CAG repeats in the androgen receptor (AR) gene and its association with fragile X syndrome and X-linked spinal and bulbar muscular atrophy, respectively, showed for the first time that expanded repeats could lead to pathology [1,2]. Nowadays more than 50 repeat expansion neurological and neuromuscular disorders have been described. Examples of repeat expansions diseases include Huntington’s disease (HD), *C9orf72*-frontotemporal dementia (FTD)/amyotrophic lateral sclerosis (ALS), myotonic dystrophy types 1 (DM1) (Figure 1) and 2 (DM2), fragile X syndrome, spinal and bulbar muscular atrophy, Friedreich ataxia, spinocerebellar ataxias and progressive myoclonic epilepsy type 1, among others (Figure 1).

The repeats length ranges from trinucleotides to the dodecanucleotide CCCCGCCCCGCG repeat of progressive myoclonic epilepsy type 1 [3]. Dominant repeat expansion diseases typically show clinical anticipation, leading to a worse form of the disease and earlier onset in the offspring [4]. This is caused by the tendency for expanded repeats to further enlarge during gametogenesis.

Triplet repeat expansions of CAG or GCN codons affecting coding regions lead to abnormally long stretches of polyglutamine (polyQ) or polyalanine (polyA) respectively within proteins. The mutant protein can exhibit a toxic gain-of-function mechanism when aggregate and accumulate in neuronal intranuclear inclusions. Pathogenic repeat expansions can also affect non-coding regions. Then, although their pathogenesis is quite variable, some common molecular mechanisms have been proposed for all of them: (1) alteration of the expression of the genes affected by the repeat motif, with protein loss-of-function or gain-of-function mechanisms operating depending on the type, length and location, (2) RNA gain-of-function mechanisms caused by the interaction of the expanded transcripts with RNA binding proteins, and (3) the production of toxic repeat peptides via repeat-associated non-ATG (RAN) translation, an unconventional translation mechanism [5]. 

The last mechanism constitutes a novel field of research in repeat expansion disorders. It involves the translation of expanded repeat RNA molecules to polypeptides without the need of a start codon. This phenomenon was first described by Zu and his collaborators back in 2011. This process can occur both at the sense and antisense strand in all three reading frames [6]. The mechanism behind it remains largely unknown, although some breakthroughs are being made in understanding it. At the time of this review’s writing, RAN translation has been described in ten repeat-expansion disorders, independently of the location of the expansion [7,8]. These include spinocerebellar ataxia types 8 [6,9,10], 31 [11] and 36 [12]; DM1 [6] and DM2 [13]; HD [14]; *C9orf72* FTD and ALS [15,16,17,18]; fragile X tremor ataxia syndrome [19,20]; fragile X premature ovarian insufficiency [21] and Fuchs endothelial corneal disease [22]. A wide variety of polypeptides can be synthetized by RAN translation in these pathologies. As such, in HD, polyA and polyS RAN products have been found [14]. However, these findings have been recently challenged by a recent study which suggests that there is no contribution of RAN translation to HD [23]). In the case of *C9orf72*, up to five dipeptides can be produced from both strands: polyGP, polyGA, polyGR, polyPA and polyPR [15,16,17,18]. Additionally, DM1 samples have shown polyQ from the antisense strand, while polyA and polyS have been found in cells transfected with plasmids containing the CAG repeat expansion of the antisense strand [6], and theoretically polyL and polyC may also be translated from the sense strand.

### 1.2. Mitochondria

Mitochondria are widely recognized as vital components for the survival of eukaryotic organisms. They are double membrane organelles involved in energy production, metabolic processes, calcium (Ca^2+^) regulation, redox balance, and apoptosis regulation [24]. Mitochondria carry their own genetic material, replicating autonomously from the host genome. In humans, mitochondrial DNA (mtDNA) encodes 13 proteins, primarily involved in oxidative phosphorylation (OXPHOS). The rest of the proteins needed by mitochondria are encoded by the nuclear genome and subsequently imported into the mitochondria. Consequently, the cooperation of both genomes is crucial for proper mitochondrial function [24]. Mitochondrial energy production relies on catabolic processes such as glycolysis and fatty acid oxidation, whose products are used by the tricarboxylic acid (TCA) cycle. The TCA cycle provides the molecules (NADH and FADH2) required to initiate the transfer of electrons from complexes I and II. Transfer of electrons through the different complexes (I to IV) of the electron transport chain (ETC) generate a proton gradient that is utilized by ATP synthase to synthesize ATP [25] in concomitance with the OXPHOS activity. While the mitochondria primary function is to generate ATP, they also produce reactive oxygen species (ROS) as a by-product of OXPHOS activity (with about 90% of ROS production being attributed to mitochondria) [26]. These include a wide variety of oxidant molecules as superoxide, hydrogen peroxide or singlet oxygen among others [27]. 

Mitochondrial function is regulated by a perfect coordination of mitochondrial biogenesis and dynamics. On the one hand, mitochondrial biogenesis involves the coordination of mtDNA and nuclear DNA proteins and is regulated by the PPAR-gamma-coactivator-1α (PGC-1α), a nuclear-encoded transcription coactivator. This protein interacts with transcription factors, such as the nuclear respiration factors 1 and 2 (NRF1 and NRF2, respectively), that upregulate the expression of mitochondrial transcription factors A (TFAM) and 2 (TFB2M), required for the replication of mtDNA [28]. A reduction of these proteins levels and activity leads to a decrease in mitochondria and has been found in several pathological processes, for example in cancer, metabolic diseases and in neurodegeneration [29].

On the other hand, mitochondrial fission refers to the scission of mitochondria into smaller organelles, while fusion involves the merging of individual mitochondria to form a single interconnected network. During fission, the protein dynamin-related protein 1 (DRP1) plays a crucial role in mediating the constriction and division of mitochondria. This process is regulated by various proteins that allow the recruitment of DRP1 to the outer mitochondrial membrane (OMM), including FIS1 and MFF [30]. In contrast, fusion is controlled by proteins such as mitofusins (MFN1 and MFN2) and optic atrophy 1 (OPA1) [30]. Dysregulation of either processes can lead to mitochondrial fragmentation or elongation, impairing mitochondrial function and cellular homeostasis.

Dysfunctional or excessive mitochondria are removed by the lysosomal degradation mechanism of autophagy. Autophagy plays a vital role in maintaining cellular balance by recycling cytosolic components such as cellular organelles and long-lived proteins [31,32]. Selective types of autophagy can be differentiated depending on the specific material to be degraded. Thus, a selective mitochondrial removal is termed mitophagy [33]. The regulation of mitophagy has been extensively studied, revealing a highly regulated process involving the recruitment of multiple proteins. The canonical initiation of mitophagy is common to other autophagic mechanisms: following autophagy induction signals the Unc-51-like autophagy-activating kinase (ULK1) complex is directed to specific sites where the autophagosome starts its formation [34]. There, it recruits another complex, VPS34/class III PI3K complex in charge of the formation of the phagophore, the precursor of the autophagosome. A third protein complex, the ubiquitin-like ATG16L1–ATG5–ATG12 complex, promotes the elongation of the phagophore. Moreover, another ubiquitin-like protein, LC3 is conjugated to the lipid phosphatidylethanolamine (PE) to form LC3-PE (also known as LC3-II), which aids in phagophore elongation and serves as a receptor for ubiquitinated targets [31]. Upon mitochondrial damage, the PTEN-induced kinase 1 (PINK1) protein is stabilized on the mitochondrial surface, leading to the recruitment of the E3 ubiquitin ligase Parkin. This initiates a cascade of phospho-ubiquitination events on the OMM, branding the mitochondria for degradation [35]. Ubiquitin receptors like optineurin (OPTN), sequestosome 1 (SQSTM1/p62) and Ca^2+^ binding and coiled-coil domain (CALCOCO2), recognise the ubiquitinated proteins present on the mitochondrial surface and interact with the autophagosome receptor LC3-II, guiding the mitochondria to the forming autophagosome [36,37]. Another mitophagy receptor recognised by LC3-II is the Bcl-2 interacting protein 3 (BNIP3), present in the OMM [31]. Autophagosomes are transported along microtubules to the perinuclear region, where they fuse with lysosomes to form autolysosomes, where mitochondria are finally degraded.

Healthy mitochondria regulate calcium level by sequestering the calcium influx from plasma membrane Ca^2+^ uptake or endoplasmic reticulum (ER) Ca^2+^ release. Ca^2+^ ions enter the OMM via the voltage-dependent anion channel (VDAC), while the inner mitochondrial membrane (IMM) needs a specific Ca^2+^ pore-forming complex: the mitochondrial Ca^2+^ uniporter (MCU) [38]. The Ca^2+^ uptake through the pore is regulated by two subunits of the MCU complex, the mitochondrial Ca^2+^ uptake proteins (MICU) 1 and 2 [39]. While some essential mitochondrial enzymes require Ca^2+^ for their functioning, an exacerbated Ca^2+^ mitochondrial concentration is responsible for the opening of the mitochondrial permeability transition pore (mPTP) and the release of cytochrome c to the cytosol, which leads to apoptotic cell death [38]. Indeed, excessive mitochondrial Ca^2+^ concentration provokes the inhibition of the anti-apoptotic proteins of the BCL-2 family (Bcl-2 and Bcl-XL). This allows the activation of pro-apoptotic factors Bax and Bak, which translocate to the OMM and form pores in the mitochondrial membranes to induce the release of several mitochondrial pro-apoptotic factors as cytochrome c, second mitochondria derived activator of caspase/direct inhibitor of apoptosis-binding protein with low pI (Smac/DIABLO) and the protease HtrA2/Omi [40]. Additionally, the Bcl-2 family also regulates the formation of the mPTP. In the cytosol, cytochrome c forms a complex with the apoptotic protease-activating factor 1 (APAF-1), and procaspase-9 to yield the apoptosome. This association activates procaspase-9, which cleaves caspase-3 and caspase-7 [41]. These caspases then cleave other effector proteins, resulting in the fragmentation of DNA and mitochondria, alterations on cytoskeleton components such as actin and the display of phosphatidylserine at the outer layer of the plasmatic membrane, a signal for phagocytes to engulf and clear these cells [41]. 

Dysfunctional mitochondria have been associated with various disorders, including neurodegenerative diseases, metabolic diseases, autoimmune disorders and cancer [24,29,38,42]. In this review, we will focus on examining the evidence linking dysfunctional mitochondria to the pathogenesis of repeat expansion diseases, specifically HD, *C9orf72* FTD/ALS and DM1. We will discuss the mechanisms that cause mitochondrial damage and compare them between these diseases. This approach will help us identify common mechanisms and address existing gaps, leading to new research approaches for repeat expansion diseases.

## 2. Discussion

While sharing common mechanisms, certain repeat expansion disorders have been better linked to a major mechanism. For this review, we selected three of them attending to their key mechanism of action. Huntington’s disease is the most well-known example of protein gain-of-function although it has also shown protein loss-of-function effects [43,44,45], as the expanded CAG repeat affects a coding region. In contrast, DM1 is the classical model of RNA toxicity, although RAN peptides have also been described [6,46]. On the other hand, RAN translation products have demonstrated a key role in C9orf72 FTD/ALS pathogenesis, however most recent literature supports a model where a combination of C9orf72 haploinsufficiency and gain of function toxic mechanisms might drive disease pathogenesis via a synergistic effect [47,48,49].

Many of the mitochondrial dysfunctions observed in these three disorders are common to them all. For example, there are alterations at different levels of the mitochondrial metabolism, specially in TCA and OXPHOS [50,51,52]. This aligns with the fact that all these repeat expansion diseases show an increase in ROS generation [52,53,54]. Mitochondrial fusion and fission processes tend to show an imbalance towards fission upregulation [44,55]. Additionally, Ca^2+^ homeostasis is deregulated in these pathologies, with a general increase in Ca^2+^ levels in all of them that leads to an exacerbated mitochondrial Ca^2+^ uptake, except in C9orf72, where a decrease in mitochondrial Ca^2+^ entry has been observed [56,57,58]. Mitophagy is also affected, with a decrease in HD and in C9orf72 FTD/ALS, while there is an increase in some autophagy markers in DM1 [43,51,59,60]. Finally, an increase in intrinsic apoptosis has been observed in all three diseases [61,62,63], possibly due to accumulated mitochondrial damage. The specific mechanisms by which these repeat expansion diseases cause mitochondrial harm will be discussed in detail further below.

### 2.1. Mitochondria in Huntington Disease

Huntington’s disease (HD) is an inherited illness caused by a dominant mutation in the hungtintin (HTT) gene, located on chromosome 4 (4p16.3). HTT codes for a 348 kDa scaffold protein which participates in a series of different processes: vesicle trafficking, cell division, ciliogenesis, endocytosis, autophagy and transcription regulation [45]. The mutation involves an abnormal repetition of a CAG trinucleotide sequence in exon 1 of HTT [64]. The CAG repeats are responsible for the production of an abnormal form of the huntingtin protein that contains an expanded polyglutamine (polyQ) tract named mutant huntingtin (mHTT). The length of the CAG repeat is inversely correlated with the age of onset of HD, with longer CAG repeats associated with earlier onset and more severe symptoms [65]. The exact mechanisms by which mHTT causes neurodegeneration in HD are complex and not yet fully understood. However, it is known to mediate toxicity through the formation of protein aggregates whithin cells. The toxic effects of mHTT aggregates result in impaired cellular functions, disrupted intracellular signaling pathways and altered gene expression. These effects lead to mitochondrial dysfunction, oxidative stress, and excitotoxicity, and other pathological processes [66]. 

The relationship between HD and mitochondrial dysfunction has been an area of active research. Subsequently, studies have shown that mHTT can directly interact with mitochondrial components (such as protein transporter TIM23), disrupting mitochondrial dynamics and impairing mitochondrial function [67,68]. Dynamic events allow mitochondria to adapt to changing cellular needs, maintain their integrity, and coordinate energy production. Mitochondrial fission and fusion are crucial processes that ensure mitochondrial dynamic and regulate the morphology, function, and homeostasis of mitochondria. In HD patients, the expression of pro-fusion proteins MFN1, MFN2 and OPA1 was downregulated, while DRP1 and FIS1 were upregulated (Figure 2) [69]. These data sustain an imbalance between mitochondrial fusion and fission in HD. The increase of mitochondrial fission in HD causes more fragmented and less motile mitochondria. This excessive mitochondrial fragmentation can be due in part to the interaction of mHTT with DRP1, [44] which significantly increases the activity of the latter. Consequently, expanded polyQ tracts induce mitochondrial morphology alteration and fragmentation [70]. These alterations were associated with a decrease in PGC-1α levels, a protein implicated in mitochondrial biogenesis and function [44]. PGC-1α regulates the expression of antioxidant enzymes that protect cells against an excessive production of ROS emanating from oxidative phosphorylation activity [71]. It has been shown that mHTT affect mitochondrial biogenesis by interacting and thus interfering with cAMP response element-binding protein (CREB) and TATA-binding protein-associated factor 4 (TAF4) (Figure 2) [72]. This leads to a reduction in PGC-1α activity that affects the antioxidant enzyme superoxide dismutase (SOD2) gene expression [72]. 

While some studies reporting mitochondrial respiration impairment did not observe evidence supporting changes in the activity of the oxidative phosphorylation (OXPHOS) complexes [50,73], others have found a decrease in their activity, particularly affecting complex II and III (Figure 2) [74,75]. Despite all of that, ROS production in HD samples was seen augmented, even before the clinical manifestations appear [53]. Moreover, studies focusing on TCA suggest a decrease in aconitase, α-ketoglutarate dehydrogenase (α-KGDH) and succinate dehydrogenase (SDH) activities in mice and human brain HD samples [76,77]. However, other research has also found an increase in SDH activity in human and mice HD cortex samples, with a rise of aconitase found only on mice [78]. Overall, the TCA cycle is not spared in HD, it being the primary source of ATP generation within cells [25]. Therefore, the alterations of enzymes involved in TCA cycle may contribute to energy deficits and metabolic disturbances in HD. 

All of these studies support an alteration in mitochondrial function. Consequently, the downregulation of mitochondrial biogenesis in HD may be the trigger for excessive mitochondrial fission. This mechanism does not appear to be compensatory during mHTT-mediated toxicity, as a partial inhibition of mitochondrial fission [44] was enough to restore a protective balance in mitochondrial dynamics. 

Alhtough mitochondrial biogenesis was reduced, an increase in mitochondrial mass was observed [43]. We hypothesized that mitophagy, a cellular process involved in the clearance of damaged mitochondria, may be altered in HD. Of note, HTT participates in the formation of the autophagy initiation complex by binding ULK1 and promoting the interaction between ubiquitin receptor p62, ubiquitinated substrates and the autophagic vesicle marker LC3-II [45]. In addition to its role in the initiation of autofagosome formation, HTT is involved in the organelle and vesicle trafficking through a direct interaction with dynein or through the hungtintin-associated protein 1 (HAP-1) (Figure 2) [57]. The association of mHTT with HAP-1 and the sequestration of other components of the trafficking process, such as microtubules or P150, has been demonstrated to impede mitochondrial trafficking in mice and in human brain samples [79,80]. Indeed, mHTT impairs the initiation complex (Figure 2), leading to a decrease in autophagy and subsequent impairment of mitophagy [43]. This is in consistence with what was observed in striatal cells, where despite the ubiquitination of depolorized mitochondria, mHTT lead to the accumulation of damaged mitochondria by precluding the recruitment of mitophagy receptors, such as OPTN and CALCOCO2, to LC3-II, which is associated with the autophagosome membrane (Figure 2) [43]. The specific mechanism by which mHTT interferes with the recruitment of mitophagy/autophagy components was not established, However, it will be interesting to investigate whether mitophagy receptors were also located in ubiquitinated damaged mitochondria. An interesting fact is that mHTT does not seem to interact with the ubiquitin system, which provide an alternative pathway independent to the Parkin protein. However, it still requires PINK1, the kinase protein implicated in ubiquitin-dependent mitophagy, which was essential for ensuring a basal mitophagy process in neuronal HD. Accordingly, overexpression of PINK1 lead to a partial removal of mitochondria in striatal cells derived from HD mice [81]. 

Together, all of these data do not distinguish whether mitochondria are a direct target of mHTT, or if mitochondrial dysfunction is a collateral damage resulting from the impairment of signaling pathways, such as Ca^2+^ signaling. In HD, aberrant calcium signaling has been implicated in the pathogenesis of the disease [61]. The mHTT protein can alters NMDA receptor (NMDAR) activity or impair the receptor signaling [82]. NMDAR is an ion-channel glutamate receptor and when activated, it allows the entry of calcium into neurons (81). In parallell, a scaffold protein named postsynaptic density-95 protein (PSD-95) interacts with NMDAR as shown in Figure 2. This interaction enables the clustering of NMDAR at the postsynaptic membrane and inhibits their negative regulator STEP61, thereby modulating their activation and signaling [83,84]. In postmortem studies and mouse model of HD, the association between NMDAR and PSD-95 is enhanced, causing NMDA-mediated exitotoxicity and neuronal death [82,85]. Moreover, mHTT can bind to inositol-3-phosphate receptors (I3PR) via HAP-1 (Figure 2), leading to the release of Ca^2+^ from the ER [86].

This increase in calcium influx compromises the capacity of Ca^2+^ uptake into mitochondria [61,87], resulting in mitochondrial membrane depolarization. Indeed, mitochondria from HD are more vulnerable to calcium overload, and mitochondrial depolarisation is preceded by a change in mitochondrial membrane potential (MMP) until the calcium burden causes the opening of the mPTP [56]. This opening leads to the release of sequestered calcium from mitochondria and subsequently triggers apoptosis in various HD models. These events are supported by the increase in cytosolic levels of Smac/DIABLO (Figure 2), which inhibits the anti-apoptotic properties of other proteins, such as X chromosome-linked inhibitor of apoptosis (XIAP) and inhibitor of apoptosis protein-1 (IAP1), in striatal cell lines expressing mHTT [88]. The binding of mHTT to p53 facilitates its nuclear translocation and, therefore, the gene expression of some pro-apoptotic factors, such as Bax [89]. Both mRNA and Bax protein levels were increased in R6/1 mice brain samples [90,91]. A study conducted in HD mice demonstrated that caspases 1, 3, 8 and 9 were progressively more active, with caspase 1 being the first to become active at 7 weeks of disease progression [91]. Also, caspase-2 can cleave mHTT, producing toxic N-terminal fragments, and caspase-7 interaction with mHTT is known to activate other caspases [72]. These events emphasize the detrimental effects of mitochondrial dysfunction, impaired autophagy/mitophagy, and the interaction with calcium signaling and apoptotic pathways in the development of HD and its neurodegenerative processes. Based on the consequences of mitochondrial dysfunction in HD, potential therapeutic strategies could target enhancing mitochondrial function and restoring energy production through effective mitophagy/autophagy activiation.

### 2.2. Mitochondria in C9orf72 Frontotemporal Dementia/Amyotrophic Lateral Sclerosis

The chromosome 9 open reading frame 72 or C9orf72 is a gene located in the short arm of chromosome 9 (9p21.2) [92]. C9orf72 was first described in 2011 following studies in familiar frontotemporal dementia (FTD) and amyotrophic lateral sclerosis (ALS) cases that described a pathogenic GGGGCC hexanucleotide repeat expansion (HRE) in the first intron of the gene. This pathogenic HRE is the primary cause of familiar forms [92,93] and is also responsible for approximatively 6% of the sporadic cases [94]. C9orf72 codes for a protein of the same name, mainly expressed in neurons, that forms part of a multimeric complex with Smith–Magenis chromosome region 8 (SMCR8) and WD40-repeat containing protein 41 (WDR41) proteins [95]. The C9orf72-WDR41-SMCR8 complex was initially proposed to have GEF activity on Rab GTPases, such as Rab8A and Rab39B, resulting in their activation [49,96,97]. However, recent in vitro studies have suggested opposing GAP activity [95,98,99]. Nevertheless, C9orf72-WDR41-SMCR8 participates in the regulation of a wide variety of processes, for example autophagy, actin dynamics, endo-exocytosis and inflammation [100]. There are three pathogenic molecular mechanisms associated to the pathogenic HRE (1) the large HRE is transcribed into sense and antisense repeat RNA which form aggregates termed RNA foci. The HRE-containing RNA transcrips may adopt different secondary structures, being one of the most characteristic RNA G-quadruplexes [101], which can interact with several RNA-binding proteins, interfering with their functions. Examples of such proteins are the heterogeneous nuclear riboprotein or hnRNP family (being the most common hnRNP H) [48], nucleolin [101], zinc finger protein 106 (Zfp106) [102] and Pur-α [103]. (2) The expanded RNA molecules can be translated in both direction into five different dipeptide-repeat proteins (DPRs), which form protein aggregates in C9orf72-ALS/FTD patient brain and spinal cord. Some of them have shown toxic properties in animal models [16,104]. (3) Finally, the HRE affects the expression of the gene provoking a reduction in C9orf72 protein and so alterations in the pathways in which the protein takes part. This loss of function mechanism has been also proposed to synergize the gain of toxicity mechanisms described above [105,106]. 

**Figure 3:**
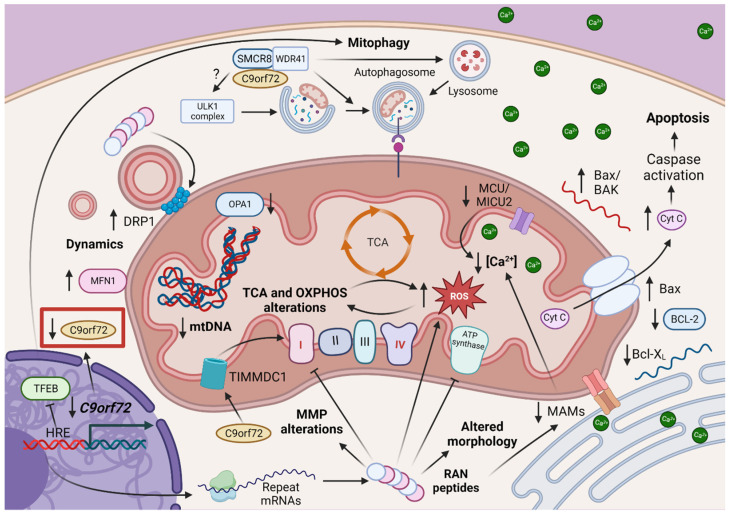
Mitochondrial alterations associated with C9orf72 HRE pathogenesis. A 50% reduction in mtDNA has been observed in C9orf72 prefrontal cortex samples. The HRE provokes a reduction in C9orf72 protein levels, which affects C9orf72 function. For example, C9orf72 prevents the degradation of mitochondrial transporter TIMMDC1, which participates in OXPHOS complex I formation. Dysfunctions in complexes I and IV have been reported in C9orf72 HRE samples. Consequently, ROS levels are increased. Ca^2+^ cytosolic levels are incremented, but defects in mitochondrial Ca^2+^ uptake associated with MCU and MICU2 decrease have been reported. HRE-containing mRNAs which reach the cytoplasm may be translated into DPR RAN peptides, affecting mitochondrial homeostasis at different levels. For example, poly-GA, poly-GR and poly-PR DPRs impair MAMs formation. Poly-GR expression alters MMP, increases DRP1 and decreases OPA1 protein levels (although MFN1 seems to be upregulated) and impairs complex I and ATP synthase activity (the latter one by favoring ATP5A1 degradation). Additionally, DPRs alter mitochondrial morphology and increase ROS levels. The reduction of C9orf72 protein alters mitophagy initiation and possibly lysosomal degradation. Moreover, expanded transcripts disrupt TFEB function. Furthermore, Bax mRNA and protein levels are exacerbated in C9orf72 samples. In addition, reduced Bcl-2 and Bcl-XL mRNA was observed, paired with higher BAK mRNA levels and cytochrome c mitochondrial release.

Despite C9orf72 recent discovery 12 years ago, there are many evidences of mitochondrial deregulation in C9orf72-mediated ALS/FTD. While in prefrontal cortex post-mortem samples of C9orf72 patients the number of mtDNA showed a 50% reduction (Figure 3) in contrast to control samples [107], mtDNA and mitochondrial mass were increased in C9orf72 patient-derived fibroblasts, due to the upregulation of PGC1-α protein levels [108]. DPRs are a crucial element of C9orf72 pathogenesis. As such, it is expected that they could interact with mitochondria. Indeed, there are many evidences that mutant C9orf72-derived DPRs, particularly those with arginine, can cause mitochondrial damage (Figure 3). For example, a study in a mouse model expressing polyGR_80_ found shorter, less motile mitochondria in cortical neurons. These changes could be explained by an increase in DRP1 and a decrease in OPA1 [52]. In contrast, C9orf72 patient-derived fibroblasts displayed elevated MFN1 protein levels [108]. 

Nevertheless, these data shows that mitochondrial dynamics in C9orf72 are altered in cortical neurons towards an increase in mitochondrial fission. Regarding this, the increase in mitochondrial biogenesis could be explained as a compensatory mechanism to replace damaged mitochondria, contrary to what we believe happens in HD [67,73]. 

C9orf72 HRE also affects mitocohondrial metabolism (Figure 3). OXPHOS complex I and ATPase activities in mice expressing polyGR_80_ were reduced. Additionally, a reduction in complexes I and IV gene expression has been reported in C9orf72-human induced pluripotent stem cells (iPSC)-derived MNs [109,110]. PolyGR can bind to ATP synthase F1 subunit alpha (ATP5A1), one component of ATP synthase complex, favoring its proteasomal degradation [52]. The reduction of complex I activity can be explained by a reduction in C9orf72 protein levels, which prevents the degradation of the translocase of the inner mitochondrial membrane domain containing 1 protein (TIMMDC1), needed for the assembly of OXPHOS complex I [111]. Dysfunctions in OXPHOS can provoke an increase in ROS generation, as observed in C9orf72 patient fibroblasts, iPSC-derived MNs transduced with a polyGR80 lentivirus and NSC34 MNs transfected with GFP-polyGR_50_ or GFP-polyPR_50_ [47,108,112]. In the latter cell model the impairment in NRF2 synthesis, an enzyme with antioxidant activity, was also reported [47]. This data confirms the hindrance of mitochondrial metabolism in C9orf72 pathogenesis, which will affect ATP production and energy-dependent celullar processes. 

A decrease in autophagic clearance of mitochondria may also contribute to the increase in mitochondrial mass observed in C9orf72-related disorders. It is important to remark the widely accepted role of C9orf72 protein in autophagy and so in the degradation of damaged/old mitochondria via mitophagy. A growing body of evidence has revealed multiples roles for the C9orf72 protein in autophagy (Figure 3); while some studies suggest a role in the activation of autophagy via the interaction of the C9orf72/SMCR8/WDR41 complex with different proteins involved in the ULK complex activation of autophagy [106,113]. On the contrary, others suggest a negative regulation of autophagy via upstream modulation of mTORC1 signalling [114,115,116]. Nevertheless, loss of C9orf72 protein has a known impact on autophagy [59]. The lysosomal localization of C9orf72 also argues for a role of the protein in autophagy degradation [117,118,119]. Recently, a study linked disruption of nucleocytoplasmic transport of the autophagy-lysosomal transcription factor TFEB caused by C9orf72-HRE with impaired autophagy. Interestingly, in this case, the effect on autophagy was not linked to haploinsufficiency of the C9orf72 protein but to the expanded transcripts [120], demonstrating that pathogenic HRE in C9orf72 alters autophagy. Consequently, C9orf72 HRE might alter mitophagy, impairing their degradation and thus promoting an increase in their numbers. However, mitochondria spared from autophagic degradation may already be damaged. Thus, their accumulation will have negative effects in ROS generation and ATP production. 

One of the main regulators of mitochondrial activity is Ca^2+^. Deregulation of mitochondrial Ca^2+^ levels disrupts correct mitochondrial metabolism and is also responsible of increased cell death [38]. Indeed, Ca^2+^ dyshomeostais have been reported in iPSC-derived MNs from patients carrying the C9orf72 mutation. These neurons showed higher Ca^2+^ cytosolic concentration than control cells, paired with a lower Ca^2+^ buffering capacity of their mitochondria [57]. These defects were linked to a decreased expression of the mitochondrial Ca^2+^ uniporter (MCU) (Figure 3) and its regulatory protein mitochondrial Ca^2+^ uptake 2 (MICU2) [57]. Likewise, mitochondria contacts with the endoplasmic reticulum (ER) regulate key cellular processes such as Ca^2+^ homeostasis or autophagy [121]. Reduced ER-mitochondria contacts have been reported in iPSC-derived cortical neurons from patients carrying pathogenic C9orf72 expansions and in affected neurons of a mutant C9orf72 transgenic mice. These studies involved the toxic DPRs (Figure 3) poly-GA, poly-GR and poly-PR in the mechanism [122]. In addition to metabolism alterations, mitochondrial Ca^2+^ levels imbalances can affect MMP, as seen in C9orf72 patient-derived fibroblasts and iPSC-derived MNs expressing polyGR80, where an increase in MMP was found [108,112] and in GFP-polyGR50/polyPR50 expressing NSC34 MNs-like cells, where a decrease in MMP was observed [47]. Interestingly, C9orf72 HRE is the only disorder of the ones reviewed in this work where an increase in cytosolic Ca^2+^ levels is not accompanied by a rise in mitochondrial Ca^2+^ uptake. We believe the loss of ER-mitochondria contacts to be the main factor impairing mitochondrial Ca^2+^ trafficking. Either way, we find this line of research of special interest, given the differences found with other repeat expansion diseases. 

However, while a reduction in mitochondrial Ca^2+^ could represent a decrease in mitochondrial-mediated apoptotic cell death, this is not what happens in C9orf72 FTD/ALS. Instead, an increase in apoptotic cell death has been widely described in different C9orf72 models, although neuronal lineage cells tend to be more susceptible to this process [62,123,124,125]. Upregulation of Bax mRNA, higher Bax protein levels (Figure 3) and cleaved caspase 3 as a result of increased p53 phosphorylation was found in iPSC-derived C9orf72 MNs [126]. Moreover, iPSC-MNs carrying the HRE exhibit reduced anti-apoptotic Bcl-2 levels and Bcl-XL mRNA, while there was an upregulation of proapoptotic BAK mRNA and elevated cytochrome c release in comparison to control iPSC-MNs [109]. Additionally, SH-SY5Y cells and mice primary cortical neurons treated with synthetic PR20 peptides showed higher p53 and Bax protein levels [127]. The upregulation of apoptosis in absence of increased mitochondrial Ca^2+^ levels could mean that, at least in C9orf72 FTD/ALS, other sources of mitochondrial damage would contribute more to the apoptotic process. 

### 2.3. Mitochondria in Myotonic Dystrophy Type 1

Myotonic dystrophy type 1 (DM1) is an autosomal dominant neuromuscular disease with mutlisystemic features caused by the expansion of a CTG trinucleotide found in the 3’ untranslated region (UTR) of the DMPK gene, located on chromosome 19q13.3 [128]. DMPK codes for a serine/threonine protein kinase involved in the correct skeletal muscle structure and function, involved in processes such as Ca^2+^ homeostasis, muscle-related gene regulation, nuclear envelope organization and myotube differentiation, while also playing a role in cardiac muscle activity and synaptic plasticity [129]. 

The three common molecular mechanisms reported for other repeat expansion diseases have also been described for DM1. Firstly, the expansion affects the levels of DMPK leading to a decrease in the protein, a serine threonine kinase involved in maintaining the skeletal muscle structure and function [130]. Secondly, mRNA molecules containing the expanded tracts can exist as aggregated intranuclear RNA foci [46]. They interact with CUG RNA binding proteins, preferentially muscleblind-like (MBNL) family members and CUG binding protein Elav-like family member 1 (CELF1). These proteins are RNA metabolism regulators, implicated mainly in alternative splicing. The union of CUG-containing RNAs to them alters their function, causing abnormal splicing pattern leading to many of the biochemical alterations observed in DM1 [46]. Thirdly, the expanded transcripts can generate up to five different homopolypeptides via RAN translation [6]. 

**Figure 4:**
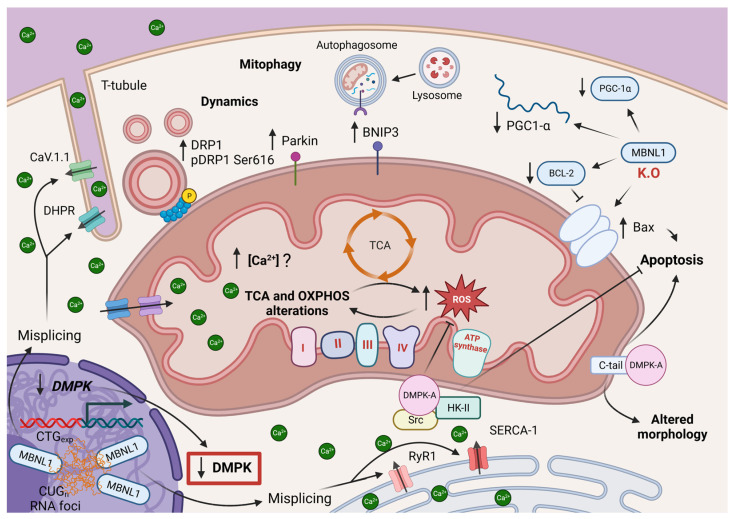
Mitochondrial alterations associated with DM1. A reduction in all OXPHOS complexes and ATP synthase gene and protein levels has been reported in DM1 muscles samples. Indeed, higher ROS generation has been found in DM1 patient fibroblasts. Elevated DRP1 and phosphor-DRP1 (Ser616) have been reported, as well as more BNIP3 and Parkin. Defects in MBNL1 splicing caused by expanded repeats mRNA molecules result in altered CaV.1., SERCA-1, DHPR and RyR1, leading to an increase in cytosolic Ca^2+^ levels which may affect mitochondrial Ca^2+^ concentration. The CTG expansion also decreases DMPK expression, meaning a reduction in its function. DMPK-A forms a complex with Src and HK-II which prevents ROS formation and protects cells from apoptosis. However, DMPK-A C-terminus tail may be responsible of altered mitochondrial morphology and mitochondrial perinuclear clustering, ultimately leading to apoptotic cell death. Finally, a study on a MBNL1 knockout model showed an increase in Bax/Bcl-2 ratio and decreased PGC-1α mRNA and protein levels.

Mitochondrial alterations in DM1 patient samples have been supported by the presence of increased mtDNA deletions [131]. The mitochondrial genes affected by these deletions are CO3 gene and ND5 gene, which encode for cytochrome c oxidase (COX) (known as complex IV) and NADH dehydrogenease subunit 5 of the complex I, respectively [131]. These two complexes are members of the OXPHOS proteins involved in the electron transport chain of mitochondrial respiration, enabling oxidative phosphorylation and ATP synthesis [27]. Therefore, mutations in one of the OXPHOS proteins could compromise mitochondrial metabolism (Figure 4), as previously reported in fibroblasts from DM1 patients [54]. 

Further evidence support an impairment of muscle oxidative metabolism in DM1 patients, which result in reduced ATP levels and compromise muscle performance [132]. Although there are no consistent data establishing a direct relation between mitochondrial mutations and metabolism impairment in DM1, gene and protein expression levels of complexes I to IV were downregulated in DM1 patients (Figure 4) and mice muscle samples [55]. We think that the decrease in inner mitochondrial membrane proteins, including Coenzyme Q10 (CoQ10), could be partly responsible for ROS generation in DM1. Interestingly, defects in mitochondrial metabolism and the increase in ROS generation observed in DM1 patient-derived fibroblasts was mitigated by metformin, an activator of AMPK [54]. Moreover, all these changes could be partially reversed by aerobic exercise [51] through the activation of AMPK pathway, which restores the level of OXPHOS proteins to that of control in DM1 [55]. Therefore, we hypothesize that DM1 models are susceptible to autophagy induction, and AMPK improves mitochondrial status by promoting PGC-1α-mediated mitochondrial biogenesis, and removal of damaged mitochondria.

Additionally, the increase of mitochondrial fission (phosphor-DRP1Ser616), autophagic and mitophagic markers (BNIP3 and Parkin) in DM1 models (Figure 4) allow us to think that basal autophagy might be triggered, but it is not enough to conduct the clearance of dysfunctional mitochondria and needs to be enhanced. It would be interesting to study whether mitochondria are being sequestered for degradation or if the degradation process is temporary delayed, as reported in DM1-derived fibroblasts [60]. 

However, efficient degradation is necessary to maintain cellular homeostasis and prevent the accumulation of potentially harmful material. A deficit in mitochondrial turnover may accelerate DM1 muscle wasting when calcium levels are disrupted. This increase in calcium is due to defects in the alternative splicing of the voltage-dependent T-tubule Ca^2+^ channel CaV.1.1 [133], the sarcoplasmic reticulum Ca^2+^ ATPase 1 (SERCA-1), the dihydropyridine receptor (DHPR) present in T-tubules and the ER ryanodine receptor (RyR1) [46]. Importantly, SERCA-1 and RyR1 are critical for calcium release and uptake in the sarcoplasmic reticulum (SR) of muscle cells. Therefore, if SR fails to pump Ca^2+^, dysfunctional mitochondrial will be unable to buffer increased cytoslic calcium levels. This can subsquently lead to damage organelles and cell death. However, further researchs are needed to establish a clear link between mitochondrial turnover and calcium homeostasis in DM1. 

Another molecular mechanism potentially related to mitochondrial alterations in DM1 could involve the loss of function of DMPK, which is caused by the presence of the pathogenic expansion [48]. Through alternative splicing, up to seven DMPK isoforms (A to G) may be synthesized, each with a common N-terminus leucine-rich domain, a kinase domain and a coiled coil domain. Variations in the C-terminus tail and the presence or absence of a VSGGG motif are responsible of the A to F isoforms of DMPK, with the exception of the human-only isoform DMPK-G, produced via the rare splicing event of a sixteenth exon [134]. Human DMPK-A is found primarily on skeletal muscle fibers, among other tissues, where it is known to bound to the OMM [135,136]. One of its roles is to protect the cells from ROS damage and apoptosis by forming a multimeric complex with the Src kinase and hexokinase II (HK-II) in the OMM (Figure 4) [135]. HK-II is the first enzyme of the glycolysis pathway and a negative regulator of the permeability transition pore (PTP) via its OMM localization [137], while Src is a tyrosine kinase able to sense ROS thanks to cysteine residues [138]. Thus, the reduction of DMPK protein levels associated to the CTG expansion may lead to an increase in ROS generation and apoptotic cell death. Additionally, a study shows that human DMPK-A, in a C-terminal tail dependent but kinase independent manner, causes mitochondrial morphology alterations and perinuclear clustering, which ultimately leads to apoptotic cell death [136]. 

MBNL1 is one the most studied proteins associated to DM1 pathogenesis. It is a nuclear protein that participates in alternative splicing regulation during skeletal muscle differentiation, affecting genes such as cardiac troponin-T (c-TNT), insulin receptor (IR) and SERCA-1, among others [139,140]. It recognizes and binds to YGCY sequences in mRNAs, allowing or repressing their processing by competition with other splicing factors [141,142]. MBNL1 is also able to interact with CUG(exp) tracts found in DM1, involving the RNA gain-of-function mechanism of the expansion. In consequence, the RNA expansions sequester MBNL1 in nuclear foci, reducing the available levels of this protein and therefore preventing its functions [46]. A recent study by Yokoyama and colleagues suggests that MBNL1 knockdown in C2C12 myotubes decreases mRNA and protein levels of mitochondrial biogenesis marker PGC-1α and increases Bax/Bcl-2 ratio (Figure 4), leading to an increase in apoptosis of these cells [63]. However, another previous research showed no changes in PGC-1α levels in DM1 patient fibroblasts [54]. These results highlight the requirement of a more extensive research.

## 3. Existing Gaps and Future Directions in Mitochondrial Dysfunction in Repeat Expansion Diseases

Taking into consideration all the data mentioned above, we consider mitochondrial alterations in repeat expansion diseases as an active area of research. However, some questions remain unanswered. For example, the role expanded RNA repeats transcripts may have on mitochondrial dysfunction in these pathologies has not been established. These RNA molecules can affect mitochondria indirectly (as we have discussed previously), but there is no data on a direct interaction between expanded RNAs and mitochondria resulting in mitochondrial damage. 

RAN translation has been described both in HD and in DM1 [6,14], but the impact of the associated RAN peptides on mitochondrial function have not been addressed. As previously described, C9orf72-derived RAN translation products are a threat to mitochondrial homeostasis. Likewise the expression of the homopolymeric repeats associated to HD and DM1, polyA and polyS in HD [14] and polyQ, polyA and polyS in DM1 [6]) have been shown to induce mitochondrial damage in several studies [143,144,145]. Nevertheless, it would be interesting to study the effects of RAN peptides on mitochondria in these diseases. 

Changes in mitochondrial dynamics have been observed in C9orf72 FTD/ALS. This was highlighted in a mice cortical neuron model by a trend towards mitochondrial fission [42], and in patient-derived fibroblasts by an increase in the pro-fusion protein MFN1 [111]. However, these differences may depend on the disease-model. Given the multisystemic nature of this disease, further research in other cell lineages is needed in order to better understand if these changes are neuronal-specific or appear in other cell types. 

As mentioned above, mitochondrial Ca^2+^ uptake is impaired in C9orf72 FTD/ALS, either by MCU and MICU2 downregulation [57] or loss of ER- mitochondria contacts [122]. Alterations in mitochondrial Ca^2+^ concentration can affect mitochondrial function, MMP and intrinsic apoptosis [38]. However, the decrease in mitochondrial Ca^2+^ does not correlate with a decrease in intrinsic apoptosis in C9orf72 models. In fact, there are numerous reports on increased apoptotic cell death in models of the disease [123,124,125]. As such, more research on which mechanism would be responsible of the rise in apoptosis in this disease is needed. Additionally, a possible therapeutic target lies in restoring normal mitochondrial Ca^2+^ uptake. Indeed, kaempferol, a natural flavonoid, has shown the ability to restore mitochondrial Ca^2+^ uptake by directly enhancing MCU activity [146]. It would be interesting to test whether kaempferol has the capacity to restore correct mitochondrial Ca^2+^ levels and if it ameliorates C9orf72 pathogenesis.

Although mitochondrial function has been studied in DM1 models, some gaps persist. The involvement of DMPK-A in preventing ROS damage and apoptosis by forming a complex with HK-II and Src at the OMM [135], together with the reduction in DMPK expression in DM1 [130] can lead to think that a lack of DMPK-A may play part in mitochondrial damage in DM1. However, DMPK-A has also shown the ability to induce mitochondrial clustering and morphological changes [136]. As such, we believe it is the lack of association between DMPK-A, HK-II and Src, while DMPK-A remains at the OMM which may be responsible for the latter effects. Also, being altered splicing one of the main features of DM1, a change of DMPK splicing leading to an increase in DMPK-A expression over other DMPK isoforms could also be responsible of mitochondrial damage in DM1. Currently, there is no data regarding isoform-specific DMPK variations in DM1, so the study of mRNA levels of the different isoforms could be an interesting topic of research.

Lastly, autophagy deregulation could play a main role in repeat expansion-associated mitochondrial deficits. Alterations in autophagy have been reported in HD, C9orf72 FTD/ALS and DM1, with a decrease in autophagic clearance in HD and C9orf72 FTD/ALS, and an increase in autophagic markers in DM1. The ultimate cause of HD and C9orf72 FTD/ALS can be narrowed down to the accumulation of aberrant proteins and/or RNA molecules; althouth these are potential autophagy substrates, the role of autophagy in RAN peptides or repeat RNA remain unclear. Different approaches have been used to increase autophagy in HD models, showing a reduction in mHTT levels and amelioration of certain motor symptoms, paired with increased survival, both in vitro and in vivo [147]. Likewise, autophagy induction by Apilimod, an inhibitor of PIKFYVE (a kinase whose activity supresses the fusion between autophagosomes and lysosomes) in C9orf72 iPSC-MNs was able to restore the formation of autophagolysosomes [106]. Additionally, a recent study showed that the treatment of a mice model carrying the HRE with metformin resulted in a decrease in RAN peptide levels and an improvement in neuropathological and behavioral phenotypes [148]. While these effects were associated to PKR inhibition, metformin is a well-known activator of AMPK signalling, one of the master regulators of autophagy induction [149]. Nevertheless, it would be interesting to address the impact of metformin on autophagy in these disorders. While autophagy induction seems to be positive for HD [147] and C9orf72-ALS/FTD [106], DM1 has been associated to abnormally induced basal autophagy [60]. Due to this, researchers have focused on inhibiting autophagy in DM1 models. Indeed, the levels of microRNA 7 (miR7), a potent autophagy repressor, are diminished in DM1 patient-derived muscle cells [150]. miR7 recovery by inhibiting miR7 repressor Mushashi-2 reduced autophagic activity and improved the pathogenic phenotype in DM1 patient-derived cell muscles [151]. However, metformin treatment in DM1 fibroblasts was able to induce a partial recovery in mitochondrial metabolism [54]. Since a delay in the degradation process of proteins has been reported in DM1 [60], it would be interesting to investigate whether this protective effect of metformin is inherent to autophagy regulation or its autophagy-inducing effect. Moreover, aerobic exercise, another well-known autophagy inducer [152], has also shown beneficial effects in DM1 patients [51,55]. Considering these contradictory results, we believe it is imperative to conduct further research on the autophagic process in DM1. This will help elucidate whether decreasing or increasing autophagy holds the key to a possible therapy for DM1.

## 4. Conclusions

Overall, mitochondria in HD, C9orf72 FTD/ALS and DM1 are affected at different levels. All three pathogenic mechanisms described for repeat expansion diseases can alter mitochondrial homeostasis [36,48]. Interestingly, in HD and in C9orf72 FTD/ALS both gain-of-function and loss-of-function mechanisms act synergistically, highlighting the complexity of these diseases and the requirement of integrating all pathogenic processes in order to fully understand these diseases. All these data demonstrate the impairment of mitochondrial metabolism in HD, C9orf72 pathogenesis and DM1 [74,106,129]. The alterations produced in dynamics, autophagy and apoptosis are also quite similar between all the diseases we reviewed, showing a trend towards augmented mitochondrial fission, impairment of mitophagy and increased apoptotic cell death [73,105,129,153]. However, it is noteworthy that while there is an increase in general Ca^2+^ levels in all of these repeat expansion diseases, mitochondrial Ca^2+^ uptake is hindered, something unique to C9orf72. Also, data on autophagy in DM1 show an increase in basal autophagy, not observed neither in HD nor C9orf72 HRE. These unshared features can suppose an interesting research approach to mitochondrial alterations in C9orf72 FTD/ALS and DM1, respectively.

Although the mechanisms of progression of repeat expansion diseases are different, the consequences on mitochondria seem to be inevitable. There is no doubt that these diseases (HD, C9orf72 FTD/ALS, and DM1) disrupt mitochondrial dynamics and biogenesis, which further accelerate their pathogenesis and progression. Understanding the underlying mechanisms of mitochondrial alterations in these disorders can provide potential therapeutic targets for all of them. However, further research is needed to unravel the intricacies of mitochondrial dysfunction and its impact on disease progression. As of today, repeat expansions diseases directly or indirectly alter mitochondrial function by targeting proteins, disrupting cellular processes or dysregulating calcium homeostasis. These identified targets harbor a therapeutic hope, on which research should focus to reduce neurodegenerative consequences.

## Data Availability

Not applicable.
